# AI conversational agents as population health infrastructure: promise, peril, and the case for public-interest design

**DOI:** 10.3389/fpubh.2026.1961045

**Published:** 2026-10-12

**Authors:** Meghna Rajan

**Affiliations:** PSG College of Arts and Science, Coimbatore, India

**Keywords:** artificial intelligence, conversational chatbots, digital public health, health equity, health misinformation

## 1 Introduction

Conversational artificial intelligence (AI) has moved from novelty to infrastructure. Rule-based chatbots that once nudged users through smoking-cessation scripts have given way to large language model (LLM)–powered agents capable of open-ended, multilingual, empathetic-sounding dialogue, available around the clock at near-zero marginal cost. Systematic and scoping reviews now document measurable effects of AI chatbots on physical activity, diet, smoking cessation, medication adherence, and mental distress across thousands of participants (1). The World Health Organization (WHO) itself has deployed a generative AI assistant, S.A.R.A.H., to test population-scale health communication (2). Taken together, these developments justify treating conversational AI not merely as a clinical or commercial curiosity, but as an emerging layer of public health infrastructure—one that sits alongside water systems, mass media, and vaccination programs as a determinant of how populations receive, interpret, and act on health information (3). This opinion argues that the digital public health community should engage conversational AI on exactly those terms: as infrastructure, not gadgetry. Infrastructure framing changes the questions we ask. Instead of “does the chatbot work?” we should ask who it works for, under what governance, and with what consequences for equity, trust, and the broader information ecosystem. I organize this argument around three tensions that recur across the recent literature: (i) genuine behavioral efficacy vs. fragile evidentiary depth; (ii) the promise of expanded access vs. the risk of a new digital divide; and (iii) the communicative power of generative AI vs. its capacity to manufacture and amplify misinformation (Table 1). I close by proposing that Sustainable Development Goal (SDG)–aligned public health institutions adopt a public-interest design standard for conversational AI, rather than leaving its trajectory to consumer technology markets alone.

**Table 1:** Summarizes how these three tensions map onto concrete implications for digital public health practice.

Tension	Evidence highlights	Population-level implication
Efficacy vs. evidentiary depth	~82% of behavior-change comparisons favorable, but only ~1/3 with moderate+ effect sizes; randomized controlled trials (RCTs) a minority (5)	Avoid premature scale-up; fund longer, larger, architecture-specific trials
Access vs. equity	Multilevel access framework spans design through sustainment (18); WHO S.A.R.A.H. built explicitly for scale (2)	Require equity-stratified evaluation before public deployment
Communication vs. misinformation	GenAI can both fact-check and propagate misinformation (17)	Mandate retrieval-grounding, provenance, and risk taxonomies (15) prior to release

## 2 Efficacy is real but shallower than it looks

The evidence base for AI chatbots in health promotion has grown quickly and, on balance, positively. A widely cited systematic review found that AI chatbots produced favorable outcomes across lifestyle modification domains, particularly physical activity, diet, and smoking cessation (4). A 2026 scoping review of 43 studies (5) reported that 82% of behavior-change comparisons trended positive, though only about a third reached moderate or larger effect sizes, and cost-effectiveness data were essentially absent. A meta-analysis focused on adolescents and young adults (1) similarly found chatbots effective for alleviating mental distress and supporting health behaviors, while noting substantial heterogeneity in intervention design and outcome measurement. Three limitations temper enthusiasm. First, most trials are short, non-clinical, adult-skewed, and rarely randomized at scale; only about a third of studies in the largest recent scoping review were randomized controlled trials (5). Second, “AI chatbot” spans an enormous range of systems, from rule-based decision trees to frontier LLMs, and pooling them obscures which architectural features actually drive behavior change (6, 7). Third, almost no study reports durability beyond a few months, leaving open whether early engagement gains represent sustained health improvement or a novelty effect. For a section whose remit is population-level promotion and prevention rather than individual clinical outcomes, this matters: infrastructure decisions built on inflated short-term efficacy claims risk premature, poorly targeted public investment (8).

## 3 Access without equity is not access

A second tension concerns reach. Proponents rightly note that AI chatbots are inexpensive to scale, available in multiple languages, and can operate where clinical workforce is scarce—attributes explicitly cited in WHO's own rationale for deploying S.A.R.A.H. as a research prototype for population health communication (9). Behavioral-access frameworks extend this logic, arguing that digital health tools must be evaluated not only on availability but on whether populations can meaningfully engage with them across design, delivery, dissemination, and sustainment stages. Yet the same structural gaps that limit telemedicine and mobile health also constrain chatbot benefit: broadband access, device ownership, digital literacy, language coverage, and trust in institutional actors are unevenly distributed within and between countries (10). Chatbots trained and validated predominantly in high-resource, English-language settings may perform less reliably for populations with the greatest unmet need—the same populations digital public health is meant to prioritize under SDG 3, 9, and 1′s emphasis on inclusive infrastructure and sustainable, resilient communities. A chatbot that reduces cost-per-contact for a health system does not automatically reduce disparities in outcomes; without equity-explicit design, deployment, and evaluation, it may simply reallocate existing advantages to those already best served by digital tools, echoing patterns already observed in AI-enabled primary care and telemedicine expansion (11).

## 4 The communicative power that cuts both ways

The third tension is the sharpest. The same generative capacity that lets an AI chatbot deliver personalized, conversational health guidance also lets it generate fluent, plausible-sounding health misinformation, and do so faster than human fact-checkers can respond. A 2025 opinion piece in this journal warned that generative AI tools function as de facto triage systems and informal health advisors in low- and middle-income countries, where their fluency can be mistaken for authority even when their outputs are unreliable. A parallel systematic review of generative AI and health misinformation cataloged mechanisms of production, propagation, and mitigation across text, image, and synthetic media, underscoring that the problem is not hypothetical but already observed at scale (12). Mitigation research is advancing in step. Retrieval-augmented generation has been used to improve the factual reliability of LLM-based COVID-19 fact-checking tools (13), and thematic-analysis pipelines have been proposed to detect and “inoculate” audiences against circulating misinformation narratives (14). A recent risk taxonomy specifically for public health LLM adoption (15) offers a structured way for practitioners to reason about failure modes before deployment rather than after. Conversational AI for vaccine communication, an earlier and instructive precedent, showed both promise for addressing hesitancy and clear risk of amplifying inaccurate claims when systems were insufficiently curated (16). The lesson across this literature is consistent: the communicative power of conversational AI is not neutral infrastructure like a water pipe; it actively shapes the epistemic environment it operates in, for better or worse, depending on design choices made well upstream of any individual conversation.

## 5 Toward public-interest design

None of this argues against conversational AI in public health; the direction of the evidence is cautiously positive, and the WHO's own experimentation with S.A.R.A.H. reflects institutional recognition that this technology will be used regardless of academic ambivalence. The argument is instead for treating conversational AI the way public health has learned, sometimes painfully, to treat other infrastructure: as a shared resource whose design choices carry population-level consequences, and which therefore warrants governance commensurate with its reach. Three concrete commitments follow. First, funders and health systems evaluating AI chatbot interventions should require reporting of architecture (rule-based vs. LLM-based, retrieval-augmented or not), population stratification by digital-access indicators, and effect durability beyond the intervention period—the absence of which currently limits comparability across the field (5). Second, deployment in low-resource or multilingual settings should be preceded by equity-focused validation rather than assumed from high-resource pilot data, consistent with behavioral-access frameworks that treat dissemination and sustainment as distinct design stages, not afterthoughts. Third, any public health–facing conversational agent should be built on retrieval-grounded, provenance-transparent architectures and evaluated against a structured risk taxonomy before release, rather than relying on *post hoc* misinformation cleanup. WHO's 2024 guidance on large multimodal models already articulates comparable principles—transparency, accountability, and human oversight—for governments and developers; the task now is operationalizing them at the level of individual chatbot deployments rather than treating them as abstract commitments.

## 6 Discussion

The digital public health field has an opportunity, still open but narrowing, to shape how conversational AI is integrated into population health promotion before deployment patterns harden into path dependency. The evidence reviewed here suggests genuine, if still shallow, behavioral benefit; genuine, if unevenly distributed, gains in access; and genuine, dual-use communicative power that can either strengthen or corrode public trust in health information depending on how it is engineered and governed. Framing conversational AI as infrastructure rather than as a discrete clinical tool has practical consequences: it shifts evaluation from short efficacy trials toward equity-stratified, durability-tested, misinformation-resilient deployment standards, and it shifts responsibility from individual developers toward the institutions, including public health agencies, research funders, and journals such as this one, that decide what counts as sufficient evidence for scale. A reasonable objection is that infrastructure-level governance risks slowing beneficial innovation, particularly in settings where any accessible health guidance outperforms none. That concern is legitimate and argues for proportionate, tiered oversight rather than uniform restriction—lighter validation for low-stakes wellness nudges, more rigorous requirements for agents addressing vaccine hesitancy, chronic disease self-management, or crisis-adjacent mental health support. What the evidence does not support is treating conversational AI's current trajectory as self-correcting. Left to consumer technology incentives alone, generative health chatbots are likely to continue optimizing for engagement and fluency rather than for population-level epidemiological benefit or equitable reach. The digital public health community is well positioned, through funding priorities, publication standards, and partnership with bodies like WHO, to redirect that trajectory toward public-interest design before it further consolidates around existing digital and geographic advantage.

## References

[1] Feng X Tian L Ho GW Yorke J Hui V. The effectiveness of AI chatbots in alleviating mental distress and promoting health behaviors among adolescents and young adults: systematic review and meta-analysis. J Med Internet Res. (2025) 27:e79850. doi: 10.2196/79850 41313175 PMC12661615

[2] van Kolfschooten H Goosen S van Oirschot J Schouten B Vajda I Willems L. Legal, ethical, and policy challenges of artificial intelligence translation tools in healthcare. Discover Public Health. (2025) 22:904. doi: 10.1186/s12982-025-01277-z 41487681 PMC12756319

[3] Wang Y Song Y Wang Y Yu L Wang J. Ethics and governance of artificial intelligence for health: guidance on large multi-modal models. Chin Med Ethics. (2024) 1001–22.

[4] Aggarwal A Tam CC Wu D Li X Qiao S. Artificial intelligence–based chatbots for promoting health behavioral changes: systematic review. J Med Internet Res. (2023) 25:e40789. doi: 10.2196/40789 36826990 PMC10007007

[5] Fu L Burns R Xie Y Shen J Zhe S Estabrooks P . The development and use of AI chatbots for health behavior change: scoping review. J Med Internet Res. (2026) 28:e79677. doi: 10.2196/79677 41604667 PMC12895150

[6] Kurniawan MH Handiyani H Nuraini T Hariyati RTS Sutrisno S. A systematic review of artificial intelligence-powered (AI-powered) chatbot intervention for managing chronic illness. Ann Med. (2024) 56:2302980. doi: 10.1080/07853890.2024.2302980 38466897 PMC10930147

[7] Schachner T Keller R Wangenheim FV. Artificial intelligence-based conversational agents for chronic conditions: systematic literature review. J Med Internet Res. (2020) 22:e20701. doi: 10.2196/20701 32924957 PMC7522733

[8] Hampsher-Monk SC Prieger JE Patwardhan S. Who is (still) smoking?. In: Tobacco Regulation, Economics, and Public Health, Volume I: Clearing the Air on e-Cigarettes and Harm Reduction. Cham: Springer International Publishing (2024). p. 31–146.

[9] Palmer A Schwan D. Digital mental health tools and AI therapy chatbots: a balanced approach to regulation. Hastings Center Rep. (2025) 55:15–29. doi: 10.1002/hast.4979 40557918 PMC12817016

[10] Banerjee P Hashim M Kumar M Mitra D Kumar A. Overcoming copyright protection difficulties in cloud settings. In: Digital Watermarking in Cloud Environments For Copyright Protection. Hershey, PA: IGI Global Scientific Publishing. p. 355–90.

[11] Elangovan K Ong JCL Jin L Seng BJJ Kwan YH Ng LS . Development and evaluation of a lightweight large language model chatbot for medication enquiry. PLoS Digit Health. (2025) 4:e0000961. doi: 10.1371/journal.pdig.0000961 40906651 PMC12410746

[12] Saeidnia F Majidi MM Ebrahimi F Mansoori S Zarei HA. Marker-trait association analysis revealed loci linked to salinity tolerance in a worldwide collection of safflower. Sci Rep. (2026). doi: 10.1038/s41598-026-56459-9 42270909 PMC13500683

[13] Li H Huang J Ji M Yang Y An R. Use of retrieval-augmented large language model for COVID-19 fact-checking: development and usability study. J Med Internet Res. (2025) 27:e66098. doi: 10.2196/66098 40306628 PMC12079058

[14] Malek S Griffin C Fraleigh RD Lennon R Monga V Shen L. Intervention in health misinformation using large language models for automated detection, thematic analysis, and inoculation: case study on COVID-19. J Med Internet Res. (2026) 28:e75500. doi: 10.2196/75500 41505350 PMC12791202

[15] Zhou J Chen AZ Shah D Schwab-Reese LM De Choudhury M. A risk taxonomy and reflection tool for large language model adoption in public health. Proc ACM Hum Comput Interact. (2025) 9:1–32. doi: 10.1145/3757544 41716439 PMC12916142

[16] Passanante A Pertwee E Lin L Lee KY Wu JT Larson HJ. Conversational AI and vaccine communication: systematic review of the evidence. J Med Internet Res. (2023) 25:e42758. doi: 10.2196/42758 37788057 PMC10582806

[17] Joseph J Jose B Jose J. The generative illusion: how ChatGPT-like AI tools could reinforce misinformation and mistrust in public health communication. Front Public Health. (2025) 13:1683498. doi: 10.3389/fpubh.2025.1683498 41080885 PMC12511027

[18] Kepper MM Fowler LA Kusters IS Davis JW Baqer M Sagui-Henson S . Expanding a behavioral view on digital health access: drivers and strategies to promote equity. J Med Internet Res. (2024) 26:e51355. doi: 10.2196/51355 39088246 PMC11327633

